# Supplementary material for: On the Baltimore Light RailLink into the quantum future
Source: Sci Rep. 2025 Aug 12;15:29576. doi: 10.1038/s41598-025-15545-0 (PMC12343960; doi:10.1038/s41598-025-15545-0)
Supplement: Supplementary file 1 — Supplementary Information. [file 41598_2025_15545_MOESM1_ESM.pdf]

Supplementary Information – On the Baltimore Light RailLink into the quantum future

Krzysztof Domino[1,*,+], **Emery Doucet**[2,3,*,+], **Reece Robertson**[4,2,3], **Bartłomiej Gardas**[1], **and Sebastian Deffner**[2,3,5]

[1]Institute of Theoretical and Applied Informatics, Polish Academy of Sciences, Bałtycka 5, Gliwice, 44-100, Poland
[2]Department of Physics, University of Maryland, Baltimore County, Baltimore, MD 21250, USA
[3]Quantum Science Institute, University of Maryland, Baltimore County, Baltimore, MD 21250, USA
[4]Department of Computer Science and Electrical Engineering, University of Maryland, Baltimore County, Baltimore, MD 21250, USA
[5]National Quantum Laboratory, College Park, MD 20740, USA
[*]kdomino@iitis.pl
[*]emery.doucet@umbc.edu
[+]These authors contributed equally.

A Details of QUBO encoding

In this Appendix we present details on the encoding of the various constraints relevant for train scheduling in QUBO form, alongside an illustrative example demonstrating this encoding for a specific scheduling instance.

A.1 Constraint encoding details

QUBO constraints are derived directly from the Integer Linear Programming model in Section II A, these are:

- The **minimal passing time** constraint: train j may pass between subsequent stations $s \to s'$ no faster than $\delta^{pass}_{s,s'}$, and must respect the minimum station stay time δ^{station} [cf. Eq. (7)]. For every train j and every pair of stations $(s,s') \in SP_j$ this constraint takes the form:

$$\sum_{t\in R_{s,j}} \left(\sum_{t'\in R_{s',j}, t'<t+\delta^{\text{station}}+\delta^{pass}_{s,s'}} x_{s,j,t} x_{s',j,t'} \right) = 0. \tag{A.1}$$

- The **minimal headway** constraint: two trains j and j' heading in the same direction must be separated in time by δ^{headway}, [cf. Eq. (5)]. For all pairs of trains $(j, j') \in H_s$ which have this headway dependency when entering station s, the constraint reads:

$$\sum_{t\in R_{s,j}} \left(\sum_{t'\in R_{s,j'}, t-\delta^{\text{headway}}<t'<t+\delta^{\text{headway}}} x_{s,j,t} x_{s,j't'} \right) = 0. \tag{A.2}$$

- The **rolling stock circulation** constraint: for two trains j and j' with j' following j which share the same rolling stock, train j' may not depart until train j arrives, followed by the minimal preparation time $\delta^{\text{preparation}}$ and the minimal stay time δ^{station} [cf. Eq. (6)]. For all pairs of trains $(j, j') \in RS_s$ that share rolling stock, the corresponding constraint is:

$$\sum_{t\in R_{s,j}} \left(\sum_{t'\in R_{s,j'}, t'<t+\delta^{\text{preparation}}+\delta^{\text{station}}} x_{s,j,t} x_{s,j',t'} \right) = 0. \tag{A.3}$$

From the definition of the time range $R_{s,j}$ from Eq. (2), we have that the outer sums in Eqs. (A.1)-(A.3) include at most $d_{\max}+1$ terms. The number of terms in the inner sums varies from 1 to $d_{\max}+1$. If we estimate that each inner sum has an average of $(d_{\max}+1)/2$ terms, then we expect there to be an average of $(d_{\max}+1)^2/2$ terms in total for each of the three constraint types given by Eqs. (A.1)-(A.3). From the discussions in Sec. II A concerning the size of the sets SP_j and H_s, we expect the number of constraints to be linear in $\#J$ and $\#S$. This statement relies on the reasonable assumption that there are few rolling stock constraints (i.e., that the sets RS_s are small).

A.2 Example instance: 2 trains, 18 variables

Having described the implementation of the different constraint types, we now present details on the model of 2 trains (corresponding to a disturbed schedule) with $d_{\max} = 2$ which requires 18 variables to encode. The solutions to this instance as computed from D-Wave experiments and IonQ simulations are presented in Fig. 7 and Fig. 8.

For this example the non-disturbed timetable is as follows:

- Train 1 is southbound, PS $\to$ MR $\to$ CS, with timetable arrival times of:

$$\begin{aligned} \tau^{\text{in}}_{1,\text{PS}} &= 14, \\ \tau^{\text{in}}_{1,\text{MR}} &= 17, \\ \tau^{\text{in}}_{1,\text{CS}} &= 32. \end{aligned} \tag{A.4}$$

- Train 2 is northbound CS $\to$ MR $\to$ PS, with:

$$\begin{aligned} \tau^{\text{in}}_{2,\text{CS}} &= 40, \\ \tau^{\text{in}}_{2,\text{MR}} &= 55, \\ \tau^{\text{in}}_{2,\text{PS}} &= 58. \end{aligned} \tag{A.5}$$

The disturbed input timetable we study here has train 1 initially delayed by 5 minutes, which places it in conflict with train 2 through the **rolling stock circulation** constraint.

A.2.1 Building the QUBO

Taking the maximal additional (secondary[1]) delay $d_{\max} = 2$, the ILP time variables from Eq. (2) for train 1 are restricted to lie between:

$$\begin{aligned} l^{\text{in}}_{1,\text{PS}} &= 19, & u^{\text{in}}_{1,\text{PS}} &= 21, \\ l^{\text{in}}_{1,\text{MR}} &= 22, & u^{\text{in}}_{1,\text{MR}} &= 24, \\ l^{\text{in}}_{1,\text{CS}} &= 37, & u^{\text{in}}_{1,\text{CS}} &= 39, \end{aligned} \tag{A.6}$$

and for train 2 between:

$$\begin{aligned} l^{\text{in}}_{2,\text{CS}} &= 40, & u^{\text{in}}_{2,\text{CS}} &= 42, \\ l^{\text{in}}_{2,\text{MR}} &= 55, & u^{\text{in}}_{2,\text{MR}} &= 57, \\ l^{\text{in}}_{2,\text{PS}} &= 58, & u^{\text{in}}_{2,\text{PS}} &= 60. \end{aligned} \tag{A.7}$$

From Eq. (10) there are 18 total QUBO variables labeled $x_{s,j,t}$, where the subscripts run over the three stations $s \in \{\text{PS}, \text{MR}, \text{CS}\}$, the two trains $j \in \{1,2\}$, and the three times possible for each variable. The ranges of the time subscripts are given by Eqs. (A.6) and (A.7), e.g., for the variables $x_{PS,1,t}$ the time index is $t \in \{19, 20, 21\}$.

The requirement that each train leaves each station once corresponds to the one-hot constraints of Eq. (14), written in terms of penalties in Eq. (16). In our example, there are six such constraints which lead to six terms of the form:

$$p_{\text{sum}} \left(\sum_{\substack{t,t' \in R_{\text{PS},1} \\ t \neq t'}} x_{\text{PS},1,t} x_{\text{PS},1,t'} - \sum_{t \in R_{\text{PS},1}} x^2_{\text{PS},1,t} \right), \tag{A.8}$$

yielding in total 54 QUBO elements.

The **minimal passing time** constraint has the following form when written in terms of a penalty, shown for the case of train 1 passing between stations PS $\to$ MR:

$$p_{\text{pair}} \sum_{t \in R_{\text{PS},1}} \sum_{\substack{t' \in R_{\text{MR},1} \\ t' < t+1+2}} \left(x_{\text{PS},1,t} x_{\text{MR},1,t'} + x_{\text{MR},1,t'} x_{\text{PS},1,t} \right), \tag{A.9}$$

there are four such equations (train 1 passing PS $\to$ MR or MR $\to$ CS, and train 2 passing CS $\to$ MR or MR $\to$ PS), which in total yield 24 QUBO elements.

The **rolling stock circulation** constraint written in terms of a penalty for the case of station CS takes the form:

$$p_{\mathrm{pair}} \sum_{t \in R_{\mathrm{CS},1}} \sum_{\substack{t' \in R_{\mathrm{CS},2} \\ t' < t+3+1}} \left(x_{\mathrm{CS},1,t} x_{\mathrm{CS},2,t'} + x_{\mathrm{CS},2,t'} x_{\mathrm{CS},1,t} \right), \tag{A.10}$$

yielding 12 QUBO elements.

The objective function which is to be minimized is the tardiness:

$$\begin{aligned} f(\vec{x}) = & \sum_{t \in R_{\mathrm{MR},1}} \frac{t-17}{2} x_{\mathrm{MR},1,t} + \sum_{t \in R_{\mathrm{CS},1}} \frac{t-32}{2} x_{\mathrm{CS},1,t} \\ & + \sum_{t \in R_{\mathrm{CS},2}} \frac{t-40}{2} x_{\mathrm{CS},2,t} + \sum_{t \in R_{\mathrm{MR},2}} \frac{t-55}{2} x_{\mathrm{MR},2,t}, \end{aligned} \tag{A.11}$$

where the vector $\vec{x}$ is shorthand for all 18 variables $x_{s,j,t}$. To translate this objective into a quadratic form suitable for a QUBO, we use the observation that $x_{s,j,t} = x_{s,j,t}^2$ since the variables are binary. The quadratic form Q comes from combining the penalty-encoded constraints of Eqs. (A.8), (A.9), and (A.10) with the objective function Eq.(A.11). In total there are 90 non-zero elements in this formulation of our two train scheduling problem.

A.2.2 Solutions

The example problem of this section is quite simple, and the resulting 18-variable QUBO is straightforward to solve with any classical solver. For the specific input data used for the disturbed input timetable where train 1 is delayed such that there is a conflict due to *rolling stock circulation* constraints, the optimal solution would be to let train 2 wait one minute at station CS, at which point the solution timetable may proceed with no conflicts. In terms of the 18 variables used for the QUBO representation, the optimal solution has

$$\begin{aligned} & x_{\mathrm{PS},1,19} = 1, \quad x_{\mathrm{MR},1,22} = 1, \quad x_{\mathrm{CS},1,37} = 1, \\ & x_{\mathrm{CS},2,41} = 1, \quad x_{MR,2,56} = 1, \quad x_{\mathrm{PS},2,59} = 1, \end{aligned} \tag{A.12}$$

and all other variables equal to zero. The value of the objective function from Eq. (A.11) is $f(\vec{x}) = 6$. This ground state is degenerate, as delaying train 2 by one minute at *PS* ($x_{PS,2,59} = 1 \iff x_{PS,2,60} = 1$) would yield the same value of the objective function and of the quadratic form.

An exhaustive enumeration of all 2^{18} possible values for the vector of variables $\vec{x}$ for this problem gives that the objective function may only take the values $f(\vec{x}) \in \{6.0, 6.5, 7.0, 7.5, 8.0\}$ when considering only feasible solutions which do not violate any constraints. This is reflected in Fig. 8b. The solutions with $f(\vec{x}) = 6.0$ or 8.0 are doubly-degenerate, as the delay at station PS is not counted in the objective.

Among these possible solutions, two example non-optimal solutions are nearly identical to the optimal solution but where one train required one additional minute to pass between CS and MR. The first of these solutions has

$$\begin{aligned} & x_{\mathrm{PS},1,19} = 1, \quad x_{\mathrm{MR},1,22} = 1, \quad x_{\mathrm{CS},1,37} = 1, \\ & x_{\mathrm{CS},2,41} = 1, \quad x_{\mathrm{MR},2,57} = 1, \quad x_{\mathrm{PS},2,60} = 1, \end{aligned} \tag{A.13}$$

and all other variables zero, giving $f(\vec{x}) = 6.5$. The second solution has non-zero variables

$$\begin{aligned} & x_{\mathrm{PS},1,19} = 1, \quad x_{\mathrm{MR},1,22} = 1, \quad x_{\mathrm{CS},1,38} = 1, \\ & x_{\mathrm{CS},2,42} = 1, \quad x_{\mathrm{MR},2,57} = 1, \quad x_{\mathrm{PS},1,60} = 1, \end{aligned} \tag{A.14}$$

and gives $f(\vec{x}) = 7.5$.

A.2.3 Spectral Properties

A complete and concrete specification of the QUBO to be minimized, formed from the combination of the constraints from Eqs. (A.8), (A.9), and (A.10) alongside the objective from Eq. (A.11), requires that specific values be chosen for p_{sum} and p_{pair}. As discussed in Section 2.2, identifying good choices for these parameters can be challenging. In this work, we have chosen to consider each problem instance with two choices of penalty values, one "overlapping" case with lower penalties chosen such that the sets of feasible and infeasible solutions overlap in energy and one "split" case with higher penalties such that there is a moderate energy gap separating the two sets of solutions. Figure S1 shows the distributions of energies for these two choices applied to the 18-variable QUBO discussed in this section. On a broad, coarse-grained level the two distributions look

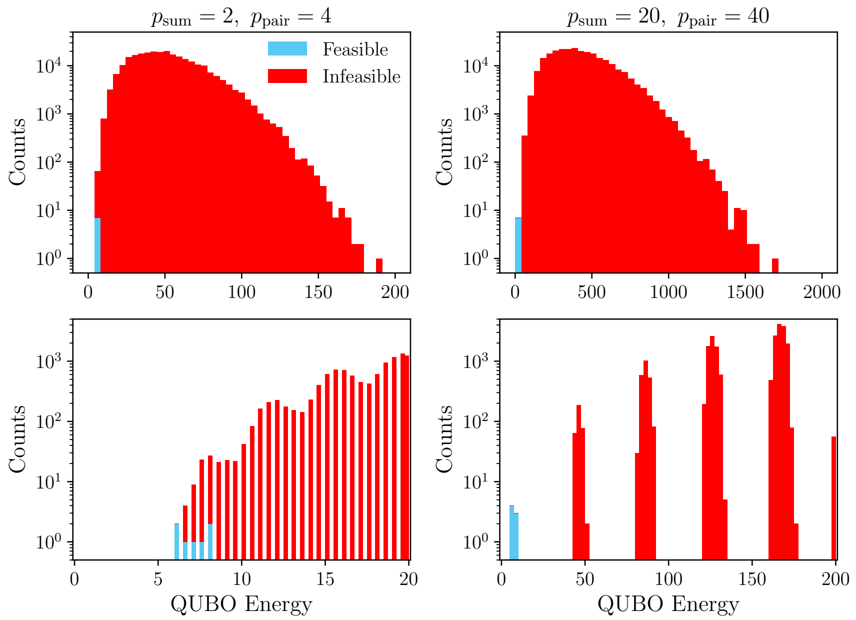

Figure S1. Histograms of QUBO values for the possible solutions to the 2 train, 18 variable problem with two choices of constraint parameters. The top panels include all 2^{18} possible solutions, and make it clear that there is little difference in the large-scale structure of the two choices beyond a rescaling of the energy. The bottom panels show only the feasible solutions as well as the infeasible solutions closest to optimal. With the lower choice of penalties, the two sets of solutions overlap, whereas with the larger penalties, the two sets of solutions are entirely separate.

essentially the same up to an overall rescaling of the energies, but the histogram of just a small window of energies around the optimal solution reveals the difference. With the smaller parameters, there is overlap between the sets of feasible and infeasible solutions and there are no large gaps (small gaps appear as all energies are integer multiples of $1/2$). With larger penalty parameters, the distribution of energies reveals clusters of solutions with similar energies separated by large gaps. These clusters correspond to different constraint violations, hence all feasible solutions are isolated in their own cluster at the lowest values of the objective.

For the larger problems we consider it rapidly becomes intractable to exhaustively enumerate the collection of solution energies, but as is clear from the results obtained with the D-Wave annealer on an 11 train, 182 variable problem shown Fig. 3 this dichotomy between overlapping and split spectra are present in all the problem instances we consider. Note that the same comb-like structure as appears in Fig. 3b and Fig. 3d would appear in the distribution of all energies in Fig. S1 if the histograms used finer binning, as in the zoomed histograms. When considering all energies a coarse binning is necessary for the feasible solution set to be visible – the D-Wave annealer is by design biased toward sampling low-energy and feasible solutions.

B Hybrid algorithm for partially-stochastic railway networks

We propose a hybrid quantum-classical algorithm to handle railway or tramway re-scheduling problems that contain a stochastic component of limited size. In the proposed approach, the stochastic component will be optimized using a quantum device which will then be used as part of a classical optimization of the deterministic component. The final output will be a set of solutions derived from various possible outcomes from the stochastic component. Practically, this technique will be useful for a variety of real-world railway (re-)scheduling scenarios such as handling worst-case scenarios due to stochasticity, simulating the impact and spread of disturbances in the stochastic component, and providing decision-makers with a series of possible solutions to choose from.

As input, the algorithm takes a description of a railway scheduling problem. This consists of a statement of the topology, parameters, and constraints relevant to the network under consideration; an initial timetable which may include disturbances; an objective function to minimize; a decomposition of the network into stochastic and deterministic components, where the stochastic component includes a small portion of the network; and some details on the statistics of train traffic through the stochastic component of the network.

From this input data, the first step is to extract the sub-problem describing only the stochastic component. Following

the approach discussed in this work, this sub-problem is transformed into a QUBO representation which can be solved on a quantum computer. Repeatedly solving this QUBO using a quantum computer or simulator produces a set of possible solutions, which can be filtered to yield a set of feasible solutions that satisfy all necessary constraints. For validation, the train traffic statistics can be computed from these solutions and compared to the specified distributions in the input data.

From this list of feasible solutions to the stochastic component some representative solutions are selected and combined with the remainder of the input data to construct a set of ILP representations of the scheduling problem on the deterministic component of the network, one per possible solution to the stochastic component. Each of these ILP problems is solved with standard classical techniques, and the best solution is determined. As it is possible that there is feedback between the stochastic and deterministic components of the network, this final solution is used in concert with the initial input data to construct a new stochastic scheduling problem. At this point, the algorithm is repeated in an iterative fashion, which may ultimately produce a joint solution with a better objective value.

Finally, the output of the algorithm is a set of conflict-free solution timetables broken down by possible behavior in the stochastic component which covers both the stochastic and deterministic components of the network.

The proposed hybrid algorithm is presented below in bullet points:

Fixed inputs

1. Problem topology, parameters, constraints;
2. Problem decomposition into stochastic (smaller) component and deterministic (larger) component;
3. Train timetable, optionally including initial disturbances;
4. Particular statistics of the train traffic on the stochastic part;
5. Objective function;

Processing

1. From initial data (timetable, disturbances), define the sub-problem on the stochastic component and encode it in QUBO form;
2. Solve the QUBO on a quantum device;
3. From QUBO solutions select feasible solutions, check the statistics of railway traffic parameters of the output, compare with expected statistics and re-sample or filter if necessary;
4. Use selected QUBO solutions as inputs to the deterministic component encoded as an ILP problem, and run multiple optimizations with multiple inputs;
5. Select a series of feasible solutions of the whole system;
6. Prepare new QUBO for stochastic component given the solution of the stochastic component in point 5, then repeat points and 2-5 check whether objective improves;
7. Execute point 6 till stopping condition.

Output

1. Series of solutions with various conflict-free timetables given various possible events in the stochastic component.

C D-Wave quantum annealer details

For our experiments with quantum annealing, we made use of the D-Wave `Advantage_system6.3` device. All the relevant physical parameters for this device are gathered in Table S1.

This device contains 5614 superconducting qubits which are coupled according to the Pegasus graph structure[3]. Since the native coupling graph of the annealer is quite far from being fully-dense, most problems must be modified to fit on the device through the use of an embedding procedure[4]. For example, one of the scheduling problems with $\#J = 2$ trains and maximal secondary delay $d_{\max} = 6$ defined in Sec. III is mapped to a QUBO with 42 variables. After translating to an Ising model and embedding into the Pegasus graph, this problem required the use of approximately 80 to 90 qubits on the D-Wave machine (the embedding procedure is non-deterministic and small variations are expected). The final embedding for this problem is illustrated in Fig. S2.

Parameter	Value
Qubits	5614
Couplers	40105
Qubit temperature (mK)	16.0 ± 0.1
Maximum achievable mutual inductance M_{AFM} (pH)	1.554
Quantum critical point (GHz)	1.281
Qubit inductance L_q (pH)	382.180
Qubit capacitance C_q (fF)	118.638
Qubit critical current I_c (μA)	1.994
Avg. single qubit thermal width (Ising units)	0.221
FM problem freezeout (scaled time)	0.073
Single qubit freezeout (scaled time)	0.616
Initial value of external flux Φ^i_{CCJJ} (Φ_0)	-0.624
Final value of external flux Φ^f_{CCJJ} (Φ_0)	-0.723
Typical readout time, one qubit to full QPU (μs)	$18.0 - 173.0$
Typical programming time (μs)	~ 14200
QPU delay time per sample (μs)	20.5
Readout error rate, full system	≤ 0.001

Table S1. Physical properties of the D-Wave Advantage_system6.3 machine[2].

The D-Wave embedding strategy uses chains of physical qubits to represent individual logical qubits. Here, we have used the default setting of *dwave.system*, the *EmbeddingComposite* Python library for all calculations on the D-Wave. The actual percentage of the number of solutions with no broken chains is greater than 50% for all calculations. In the worst case ($\#J = 11$ trains, maximal secondary delay $d_{\max} = 6$, and annealing time 10μs) we had still 55% of solutions without chain break.

D IonQ quantum computer details

Parameter	Aria-1	Brisbane
Qubits	25	127
T_1	100s	231.12μs
T_2	1s	145.97μs
Single-qubit gate time	135μs	
Single-qubit gate error	0.040%	0.025%
Two-qubit gate time	600μs	660ns
Two-qubit gate error	4.250%	0.742%
Reset time	20μs	
Readout time	300μs	4μs
SPAM accuracy	99.520%	
Readout error		1.320%

Table S2. Characterization of the two gate-based quantum processors used in this work: IonQ's Aria-1 trapped-ion device[5] and IBM's superconducting Brisbane device[6]. The reported errors and lifetimes are average values for the Aria-1 device and median values for the Brisbane device.

As a representative of the gate-based approach to quantum computing, we chose to make use of the IonQ Aria-1 device. This is a gate-based quantum computer with 25 trapped Ytterbium ions acting as qubits, supporting all-to-all connectivity (meaning, two-qubit gates are directly supported between any pair of qubits). The various parameters which characterize the performance of the Aria-1 device are shown in Table S2. Note that the average gate errors are subject to significant variation over time, though they generally appear to be within a factor of $2\times$ in either direction of the numbers reported in the table.

Finding the solution to a QUBO problem with the Aria-1 device with QAOA requires transforming the QUBO into a Ising model and finally into a circuit representing the QAOA ansatz which can be run on the device as part of a classical optimization loop. We used the Qiskit implementation of QAOA for this work, which transformed our input problems into circuits in terms

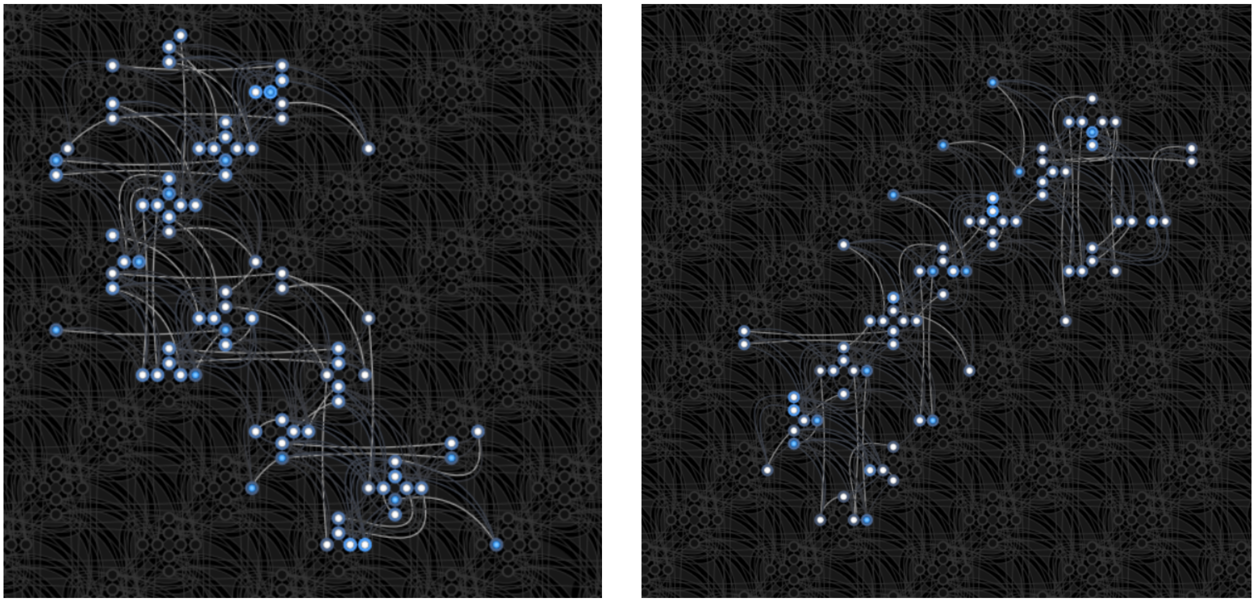

Figure S2. Illustration of the embedding of a two train scheduling problem with maximal secondary delay $d_{\max} = 6$, requiring 42 variables into the native graph of the D-Wave annealer, for both disturbed (left) and non-disturbed (right) scenarios. After embedding, roughly 80 qubits were required. Dots represent active qubits and edges active coupling between qubits.

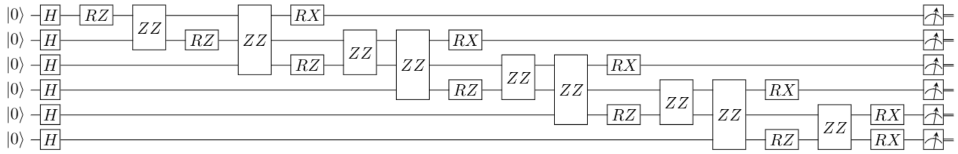

Figure S3. A quantum circuit diagram showing the single-layer QAOA ansatz for the simplest 6-variable QUBO, requiring 9 two-qubit gates (ZZ gates couple the outermost qubits the boxes cover) and a circuit depth of 17. This circuit is expressed in terms of an abstract gate set, and must be transpiled into the native gate set for any particular quantum processor for execution. The initial layer of Hadamard gates prepares the ground state of the initial Hamiltonian, then the layer of RZ and ZZ gates corresponds to the evolution under the cost Hamiltonian, and finally the layer of RX gates correspond to the mixer Hamiltonian.

of an abstract gate set which are then submitted to IonQ to be transpiled into the native Aria-1 gate set and run on the hardware.

Unlike with the D-Wave machine, since the Aria-1 device has all-to-all connectivity there is no embedding overhead and thus the number of binary variables in the original QUBO is the number of qubits required. For the smallest problem we considered, with one train and $d_{\max} = 2$, this number is six. The circuit implementing the single-layer QAOA ansatz for this problem is illustrated in Fig. S3. In this circuit, the number of two-qubit gates (depicted as ZZ) required scales with the number of couplings in the Ising model (essentially, the number of off-diagonal non-zero matrix elements in the original Q matrix defining the QUBO). The required circuit depth is not obvious from the figure, as we do not know the actual circuit run on the device after transpilation and so do not know if there are additional barriers or constraints to parallelism introduced at that stage. Nonetheless, we show in Fig. S4 the two-layer QAOA ansatz for the same problem in the abstract gate set. The number of two-qubit gates is exactly doubled since the circuit consists of two copies of the same subcircuit run one after another, however the figure shows that the required circuit depth could be significantly less than double. Since two-qubit gate errors are very likely the dominant error mechanism for the execution of these circuits this has little effect on the overall error, though it does indicate that the runtime need not double.

E Results with a superconducting IBM device

Another representative of the gate-based approach to quantum computing is provided by the superconducting quantum computers from IBM. Several of their 127-qubit `Eagle r3` devices are available through the IBM Quantum Platform, for example the Brisbane device whose properties are summarized in Table S2.

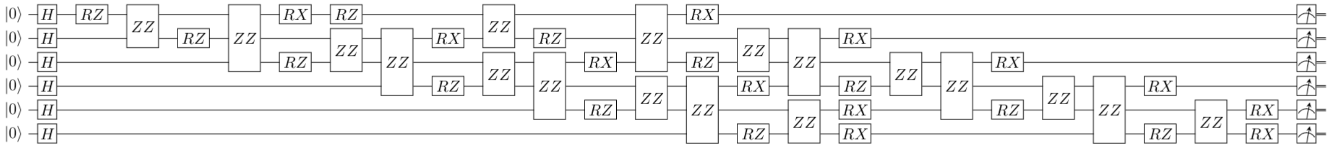

Figure S4. A quantum circuit diagram showing the two-layer QAOA ansatz for the simplest 6-variable QUBO, again in terms of an abstract gate set. This circuit contains 18 two-qubit gates and has a depth of 25.

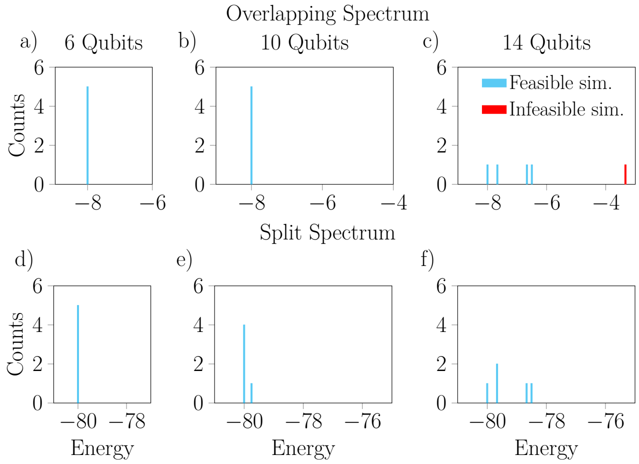

Figure S5. Histograms of the result energies yielded by noisy simulations of the IBM Brisbane superconducting gate-based quantum computer running QAOA with a single-layer ansatz. Each QUBO was minimized five times.

In principle, solving our scheduling problems on these devices should follow essentially the same procedure as with solving them on the IonQ Aria-1 device: transform the QUBO into an Ising model, then build the QAOA ansatz from the Ising model in terms of the abstract gate set which is then compiled into the native gate set supported by the hardware. One source of complexity comes from the fact that on these devices, it is not possible to perform two-qubit gates between arbitrary qubits as the qubit connectivity graph is planar and only allows two-qubit gates between adjacent qubits. Effective gates between spatially-separated qubits must involve a series of qubit-qubit swaps until the gate can be performed between neighboring qubits. In this case, since the device has a highly-constrained connectivity graph it could be advantageous to follow some form of embedding procedure whereby one logical variable is spread over multiple qubits to allay connectivity issues, as is necessary on the D-Wave quantum annealer. We did not pursue this idea here, especially since the small scheduling problems examined are likely to not benefit.

Unfortunately, due to recent changes to how the IBM Quantum Platform operates and to the requirements which must be satisfied by circuits submitted to the cloud interface, the Qiskit implementation of QAOA we have used in this work can not currently be run on IBMs hardware[7]. Therefore, we can only present results from the noisy simulator which was provided as part of the platform until recently[8]. Using the `ibmq_qasm_simulator` configured to emulate the noise characteristics of the Brisbane device, we ran each of the three smallest scheduling problems (corresponding to 6, 10, and 14 variable QUBOs) five times, with two choices of penalty values yielding an overlapping or split spectrum. In terms of the abstract gate set, the circuits submitted were essentially the same as the ones used on the Aria-1 device, e.g. as in Fig. S3 for the 6 variable problem. Unlike with the Aria-1 device, however, it is likely that the transpilation process would add additional two-qubit gates over the minimum number set by the Q matrix due to the restricted connectivity of the IBM device. For circuits with as few qubits as the ones we examine this is likely not a serious problem but for larger instances this overhead may dominate.

The distributions of final energies returned from the noisy simulator are plotted in Fig. S5. While it is impossible to draw reliable conclusions from so few data points, comparing these results to the equivalent results from the IonQ Aria-1 simulator seem to indicate that the performance of the IBM device should be expected to be comparable to the IonQ device, assuming that the simulator accurately captures the behavior of the device.

References

1. D'Ariano, A., Pacciarelli, D. & Pranzo, M. A branch and bound algorithm for scheduling trains in a railway network. *Eur. J. Oper. Res.* **183**, 643–657, DOI: https://doi.org/10.1016/j.ejor.2006.10.034 (2007).
2. D-Wave Systems Inc. QPU-Specific Physical Properties: Advantage_system6.3 (2023). Visited at 17.10.2023.
3. Dattani, N., Szalay, S. & Chancellor, N. Pegasus: The second connectivity graph for large-scale quantum annealing hardware. *arXiv preprint arXiv:1901.07636* DOI: https://doi.org/10.48550/arXiv.1901.07636 (2019).
4. Cai, J., Macready, W. G. & Roy, A. A practical heuristic for finding graph minors. *arXiv preprint arXiv:1406.2741* DOI: https://doi.org/10.48550/arXiv.1406.2741 (2014).
5. IonQ. IonQ Cloud Console. https://cloud.ionq.com (2024). Accessed: 29.4.2024.
6. IBM. IBM Quantum Dashboard. https://quantum.ibm.com (2024). Accessed: 11.6.2024.
7. ISA circuit support for latest runtime. https://github.com/qiskit-community/qiskit-algorithms/issues/164 (2024). Accessed: 14.6.2024.
8. IBM. Migrate from cloud simulators to local simulators. https://docs.quantum.ibm.com/api/migration-guides/local-simulators (2024). Accessed: 14.6.2024.
